# Effects of Strontium Modification on Corrosion Resistance of Al-Si Alloys in Various Corrosive Environments

**DOI:** 10.3390/ma17194923

**Published:** 2024-10-09

**Authors:** Lau Lin Jie, Mirza Farrukh Baig, Ervina Efzan Mhd Noor

**Affiliations:** Centre for Manufacturing and Environmental Sustainability, Faculty of Engineering and Technology, Multimedia University, Bukit Beruang, Malacca 75450, Malaysiafarrukhbaig26@gmail.com (M.F.B.)

**Keywords:** corrosion, Al-Si alloy, strontium modification

## Abstract

This study investigates the impact of strontium (Sr) additions on the corrosion resistance of an LM6 (A413) aluminium alloy. By incorporating varying concentrations of Sr (0.01 wt.% and 0.05 wt.%), the morphological and corrosion behaviours of the alloy were analysed under different corrosive environments, including sulphuric acid, sodium hydroxide, and sodium chloride solutions. The results demonstrate that Sr modifications significantly enhance the alloy’s corrosion resistance, with the most substantial improvement observed at 0.05 wt.% Sr. The analysis revealed that the weight loss of the alloy in sulphuric acid decreased by 2.5% with 0.05 wt.% Sr after 10 days of immersion, due to the formation of a stable passive oxide layer. In sodium hydroxide, however, the weight loss was reduced by 5% with 0.05 wt.% Sr after 10 days, indicating aggressive uniform corrosion. In the 3.5% sodium chloride solution, the corrosion rates remain relatively low, and the 0.05 wt.% Sr alloy showed a decrease in corrosion product formation over time, suggesting enhanced resistance. Detailed surface analyses, including 3D profiling and morphology assessments, revealed that Sr additions refine the eutectic silicon phase, transforming it from a coarse to a more desirable fibrous or lamellar structure, thus improving the alloy’s overall performance. The innovative findings underscore the potential of Sr as an effective microstructural modifier for enhancing the durability and longevity of Al-Si alloys in corrosive environments.

## 1. Introduction

Aluminium-silicon (Al-Si) alloys are widely recognised for their excellent castability and mechanical properties, making them preferred in industries such as automotive and aerospace [1,2,3]. Aircraft, with their intricate structures and demanding operational environments, are subject to various forms of corrosion that can compromise their safety, reliability, and longevity. The corrosive agents encountered during manufacturing, operation, and maintenance pose significant challenges to the structural integrity of aircraft components, particularly those constructed from aluminium alloys. Corrosion-induced damage necessitates costly repairs and replacements and undermines the operational efficiency and safety of the aircraft.

In response to these challenges, extensive research efforts have been directed towards understanding the corrosion mechanisms affecting aluminium alloys and developing effective mitigation strategies. One promising avenue of investigation lies in the modification of aluminium alloys through the addition of dopants, which can alter the alloy’s microstructure and enhance its resistance to corrosion. By strategically incorporating dopants into aluminium alloys, scientists and engineers aim to bolster their performance and durability in corrosive environments.

Numerous researchers have investigated dopant modification in Al-Si alloys like sodium (Na) [4], titanium (Ti) [5,6], boron (B) [7,8], strontium (Sr) [9,10,11], vanadium (V) [12], cerium (Ce) [13,14], beryllium (Be) [15], manganese (Mn) [16,17], and chromium (Cr) [18]. These dopants typically alter the microstructure and mechanical, chemical, casting, and heat properties [19]. However, it is crucial to acknowledge that introducing dopants may potentially compromise other alloy properties, necessitating careful consideration before implementation.

Sr and Na are the most effective eutectic modifiers, commonly used in foundry industries. However, Sr has distinct advantages over Na, including lower volatility, high recovery rate (80–90%), ease of addition, stability in the melt during holding times, production of smoother castings, non-reactivity with refractory materials, and environmental friendliness [19,20]. Sr modification in Al-Si alloys significantly impacts their corrosion resistance and mechanical properties. Research indicates that the addition of Sr refines the microstructure, reduces the size of dendrites, modifies eutectic phases, and enhances corrosion resistance in various corrosive environments [10,20,21]. The presence of Sr alters the silicon shape from a circular to spherical form, leading to improved mechanical properties such as increased ultimate tensile strength, yield strength, and hardness [9]. Moreover, the corrosion behaviour of Al-Si alloys is notably influenced by the modified eutectic phase resulting from strontium addition, with the corrosion resistance being highest in alloys with optimal Sr content [22]. Additionally, the combination of Sr grain modification and heat treatment enhances the tribo-mechanical properties of Al-Si alloys, leading to superior tribological performance under varying loads and sliding distances. Small amounts (200 ppm) of Sr modify the eutectic Si phase in Al-Si casting alloys, transforming the morphology from coarse plate-like to fine fibrous networks [23]. Another study investigated the effects of Sr modifier and various heat treatments on the wear–corrosion properties of A356 alloy, aiming to enhance wear resistance and corrosion resistance [24]. The study explored how the addition 200 ppm of Sr modifier and heat treatment influenced the morphology of eutectic Si particles, improving wear and wear–corrosion properties of A356 alloy. The addition of 250 ppm of Sr to Al-9Si alloys was shown to increase the area of eutectic phases from 26.1% to 33.0%, while reducing corrosion resistance due to galvanic coupling in a 3.5% NaCl solution [25]. However, another study [26] reported beneficial effects of Sr addition on the corrosion behaviour of A356 alloys in the same solution.

The incorporation of Sr and specific heat treatments significantly impacts the wear-corrosion properties of the Al-7Si-0.3Mg alloy. Sr modification, whether in the form of AlSr10 or AlTiBSr master alloys, leads to structural improvements such as silicon spheroidisation and grain refinement, enhancing mechanical properties like increased elongation and yield strength [27]. Additionally, the presence of strontium in the alloy affects the precipitation of finer β″-Mg_2_Si particles during heat treatment, resulting in higher tensile strength and electrical resistivity, while also providing resistance to overageing [28]. Furthermore, heat treatments play a crucial role in altering the microstructure of the alloy, consequently influencing its corrosion behaviour in NaCl solutions, with variations in treatment conditions leading to different corrosion properties, where the peak ageing condition exhibits the worst corrosion properties compared to solution treatment [29]. The influence of modification on the microstructure and corrosion behaviour of AlSi7Mg0.3 alloy was investigated and highlighted the significant impact of magnesium, Sr, and Be modifiers on the corrosion behaviour of AlSi7Mg0.3 alloy [30]. Magnesium and modifiers based on Sr and Be influenced the corrosion behaviour in a neutral corrosion fog, leading to increased pitting and overall corrosion effects.

The primary focus of this study is to evaluate the effects of Sr additions on the corrosion resistance of Al-Si alloys. By comparing the performance of an LM6 alloy with those modified with 0.01 wt.% and 0.05 wt.% Sr, this research aims to identify the optimal Sr concentration for enhancing alloy performance in various corrosive environments. The corrosion agents used in this study—sulphuric acid, sodium hydroxide, and sodium chloride—simulate typical environmental conditions that these alloys might encounter in real-world applications.

## 2. Material and Methods

In this study, LM6 (A413) aluminium alloy is used, renowned for its resistance to cracking and excellent castability, making it a popular choice in the automotive and aerospace industries [31]. The chemical composition of LM6 (A413) aluminium alloy by weight percentage is detailed in Table 1 [32].

Al-12Si-0.02Sr-0.02Na.

**Figure 1:**
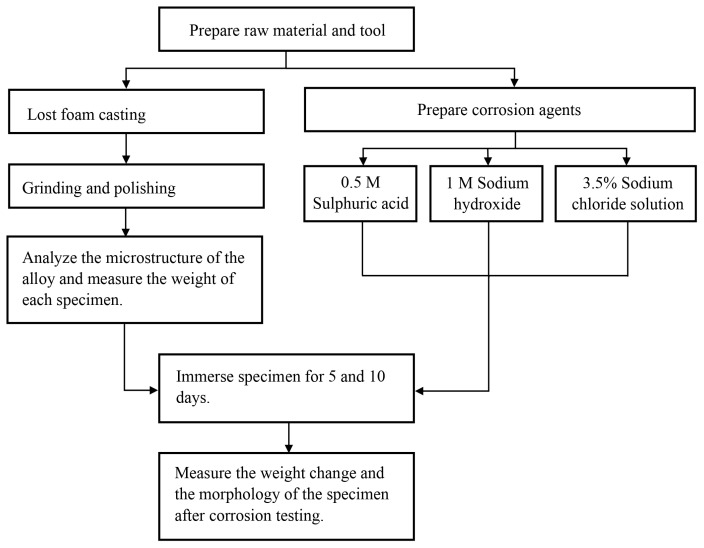
Flow chart of the experiment.

The experimental setup comprises two distinct phases: the fabrication of specimens and the preparation of corrosion agents as illustrated in Figure 1. Specimens are produced using the lost foam method, resulting in three batches: LM6, LM6 + 0.01 wt.%. Sr, and LM6 + 0.05 wt.%. Sr. Subsequently, corrosion agents, including sulphuric acid, sodium hydroxide, and a 3.5% sodium chloride solution, are prepared. Each batch of specimens is then subjected to immersion in the three different corrosion agents for two durations: 5 and 10 days. Following the corrosion tests, the surface of the specimens is examined using an optical microscope to observe any corrosion-induced changes.

### 2.1. Lost Foam Casting and Strontium Modification

For lost foam casting, meticulous attention is paid to the preparation of green sand and the crafting of polystyrene foam moulds to facilitate the creation of uniform specimens. The LM6 aluminium silicon alloy, supplemented with precise amounts of Sr modifiers, is then melted and poured into the moulds. Following solidification, specimens undergo meticulous cutting, drilling, and polishing to prepare them for corrosion testing. Each specimen’s weight is carefully recorded, and they are organised in labelled zipper bags for identification. Subsequently, the specimens are subjected to immersion in various corrosive solutions, suspended by copper wires to maintain the integrity of the testing environment. This comprehensive approach ensures that the effects of corrosion on the aluminium alloy specimens are accurately evaluated under controlled conditions.

### 2.2. Preparation of Corrosion Agents

Sulphuric acid (H_2_SO_4_) and sodium hydroxide (NaOH) solutions are diluted with 200 mL of distilled water each, resulting in solutions with pH values of 2 and 12, respectively. The accuracy of these pH values is confirmed using Merck pH strips (Merck KGaA, Darmstadt, Germany). Additionally, saltwater is prepared by mixing 200 mL of distilled water with 7 g of sodium chloride (NaCl). This meticulous preparation ensures the reliability and accuracy of the corrosion testing environment.

After exposure to the corrosive agents, each specimen undergoes meticulous cleaning and drying to prevent any further corrosion. Subsequently, the weight of each specimen post-corrosion test is carefully measured and recorded. The weight loss of each specimen is calculated using Equation (1). This systematic approach ensures accurate assessment and comparison of the corrosion effects on the specimens, providing valuable insights into their corrosion resistance properties.

(1) Weight loss=Final weight−Original weight 

(2) $$\mathrm{Corrosion}\;\mathrm{rate}=\frac{8.76\;\times {\;10}^{4}\;\times \;W}{A\;\times \;T\;\times \;D}$$

The corrosion rate can be calculated using Equation (2) [33], where 8.76 × 10^4^ is the constant used to calculate the corrosion rate in units of mm/year. In Equation (2), W represents the weight loss in grams, A is the total surface area in cm^2^, T stands for the duration of experiment in hours, and D denotes the density of the specimen in g/cm^3^.

## 3. Results and Discussion

### 3.1. Microstructure and Morphology of Modified and Unmodified LM6

**Figure 2:**
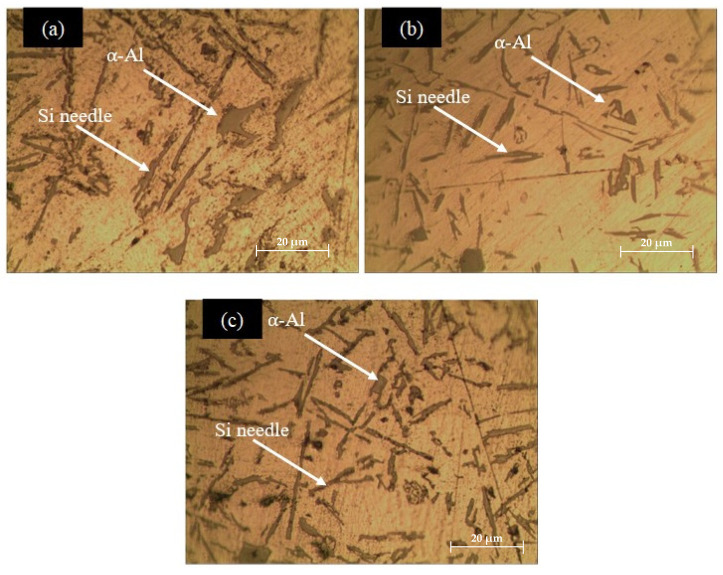
Microstructure of LM6 under 20× magnification. (**a**) LM6 alloy, (**b**) LM6 + 0.01 wt.% Sr, and (**c**) LM6 + 0.05 wt.% Sr.

Figure 2 shows the microstructure of LM6 with different wt.% of Sr. In Figure 2a, the LM6 alloy exhibits a dendritic structure typical of cast aluminium-silicon alloys. The dendrites appear elongated and interconnected, with primary aluminium (α-Al) phases distributed throughout. The eutectic silicon phase appears as dark, needle-like particles dispersed in the aluminium matrix. This morphology is expected in cast LM6 alloys due to the natural solidification process, which leads to the formation of these dendritic and eutectic structures. The addition of 0.01 wt.% Sr significantly modifies the microstructure of the LM6 alloy, as shown in Figure 2b. The eutectic silicon phase becomes finer and more uniformly distributed compared to the unmodified LM6 alloy. The needle-like silicon particles seen in Figure 2a are now shorter and less interconnected, resulting in a more fibrous or lamellar structure. This transformation occurs because Sr acts as a microstructural modifier, disrupting the formation of coarse silicon platelets and promoting a refined, uniform distribution. The refined silicon structure enhances both mechanical properties and corrosion resistance by reducing stress concentrations and corrosion initiation sites, thereby improving the alloy’s overall durability. Additionally, the primary aluminium dendrites become more uniform and less elongated, contributing further to the improved microstructure.

With the addition of 0.05 wt.% Sr in Figure 2c, the LM6 alloy shows a further refined microstructure. The eutectic silicon phase is even more finely dispersed and appears as small, rounded particles rather than needles. The primary aluminium dendrites are further refined and more uniformly distributed. This higher concentration of strontium enhances the modification effect, resulting in a very fine, well-distributed silicon phase. This refined microstructure is beneficial for improving the mechanical properties and corrosion resistance of the alloy, as it reduces stress concentrations and enhances the alloy’s overall performance.

### 3.2. Corrosion Effects of Modified and Unmodified LM6 Alloys

**Figure 3:**
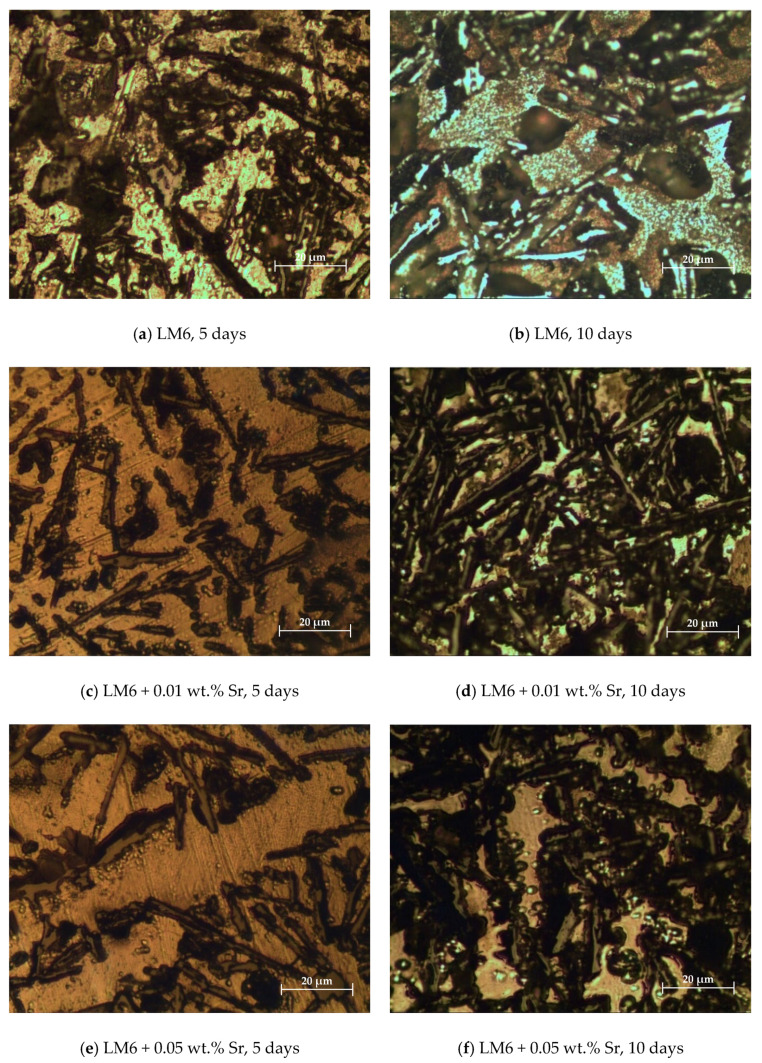
Morphology in 20× magnification immersed in sulphuric acid. (**a**) LM6, 5 days; (**b**) LM6, 10 days; (**c**) LM6 + 0.01 wt.% Sr, 5 days; (**d**) LM6 + 0.01 wt.% Sr, 10 days; (**e**) LM6 + 0.05 wt.% Sr, 5 days; and (**f**) LM6 + 0.05 wt.% Sr, 10 days.

Figure 3a–d illustrate the morphology of the modified and unmodified LM6 alloys after immersion in sulphuric acid for 5 and 10 days. In Figure 3a, the surface exhibits significant corrosion with visible pitting and roughness. The needle-like eutectic silicon phase remains identifiable, but the aluminium matrix shows heavy corrosion, indicating an aggressive attack by the sulphuric acid. After 10 days, as shown in Figure 3b, corrosion has progressed further, with extensive pitting and increased roughness, and larger areas of the aluminium matrix are eroded. The corrosion products are more pronounced, and the integrity of the microstructure is significantly compromised.

The addition of 0.01 wt.% Sr appears to refine the microstructure, making the silicon particles more fibrous and reducing the severity of pitting compared to the unmodified LM6. The surface is smoother, indicating better corrosion resistance with this modification. While corrosion is evident, the extent of pitting and roughness is less severe than in the unmodified alloy. The refined microstructure with finer silicon particles contributes to enhanced resistance to sulphuric acid corrosion over a longer period.

With the addition of 0.05 wt.% Sr, the surface morphology shows further refinement of the silicon particles, which are now even finer and more uniformly distributed. Corrosion is present but is less aggressive than in the unmodified and 0.01 wt.% Sr-modified alloys. The surface smoothness suggests improved corrosion resistance. After 10 days, the alloy with 0.05 wt.% Sr exhibits the least amount of corrosion damage among all samples. The surface remains relatively smooth with minimal pitting, and the fine, uniformly distributed silicon particles and refined aluminium matrix demonstrate significant resistance to sulphuric acid corrosion.

Overall, the addition of Sr, particularly at 0.05 wt.%, significantly enhances the corrosion resistance of the LM6 alloy, demonstrating the effectiveness of Sr as a microstructural modifier in reducing the aggressiveness of corrosion attacks. Figure 3 illustrates this progression of corrosion over time. The unmodified LM6 alloy shows significant corrosion after 5 days, with surface roughness and pit formation becoming more pronounced by 10 days. In contrast, the LM6 alloy with 0.01 wt.% Sr exhibits improved initial corrosion resistance, with a smoother surface and fewer pits after 5 days, though some degradation is still apparent after 10 days. The most notable improvement is observed in the LM6 alloy with 0.05 wt.% Sr, which maintains a smooth surface with minimal corrosion even after 10 days. This indicates that 0.05 wt.% Sr provides the best long-term protection, making it a valuable addition for enhancing the performance and durability of Al-Si alloys in corrosive environments.

**Figure 4:**
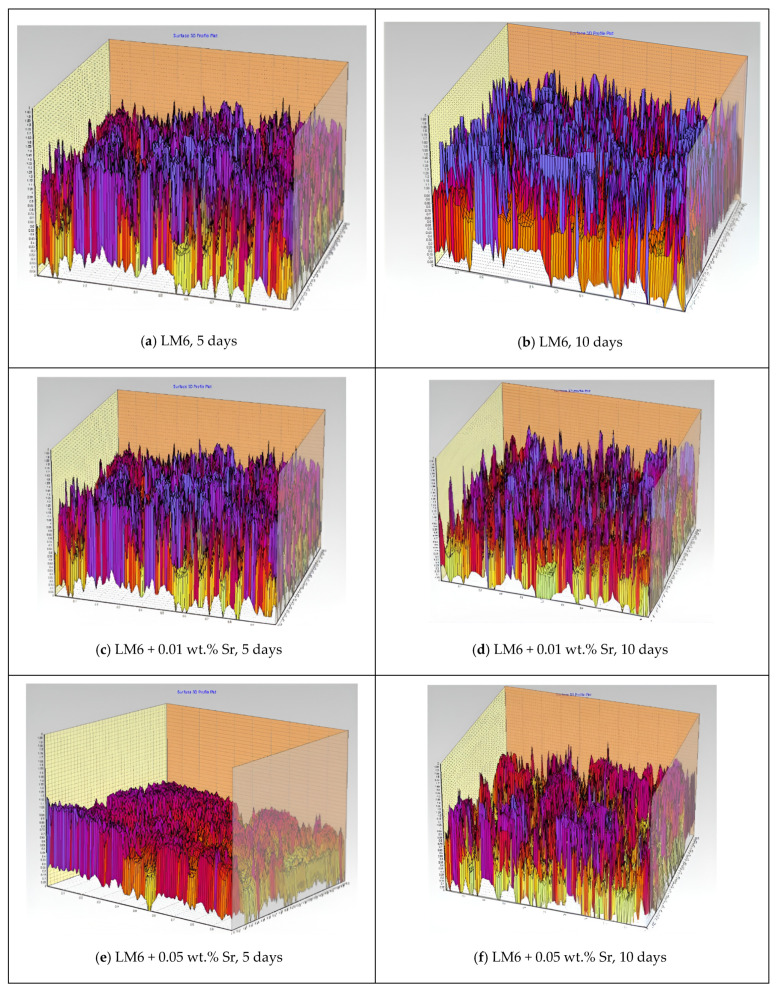
Three-dimensional profiles in 20× magnification immersed in sulphuric acid. (**a**) LM6, 5 days; (**b**) LM6, 10 days; (**c**) LM6 + 0.01 wt.% Sr, 5 days; (**d**) LM6 + 0.01 wt.% Sr, 10 days; (**e**) LM6 + 0.05 wt.% Sr, 5 days; and (**f**) LM6 + 0.05 wt.% Sr, 10 days.

Figure 4a–f present 3D profiles illustrating the surface topography of LM6 and its Sr-modified variants immersed in sulphuric acid. The colours and peaks represent surface roughness and topography over time. Higher peaks and brighter colours (e.g., red and yellow) indicate regions of greater surface roughness and more pronounced corrosion damage, while lower peaks and cooler colours (e.g., purple and blue) suggest smoother surfaces with less corrosion.

Figure 4a shows the unmodified LM6 alloy after 5 days of immersion, revealing significant surface roughness with pronounced peaks and valleys, indicating severe corrosion with deep pits and a rough corroded surface. In Figure 4b, the surface roughness has increased after 10 days, with more pronounced peaks and deeper valleys, demonstrating continued and aggressive corrosion and extensive surface degradation.

Figure 4c shows a reduction in surface roughness for the alloy modified with 0.01 wt.% Sr, with less pronounced peaks and valleys, indicating improved corrosion resistance and a smoother surface. After 10 days, the surface roughness remains moderate, with peaks and valleys present but less severe than in the unmodified alloy, showing the continued beneficial effects of the 0.01 wt.% Sr modification.

Figure 4e reveals that the surface is notably smoother for the alloy modified with 0.05 wt.% Sr compared to both the unmodified LM6 and the 0.01 wt.% Sr-modified alloy. The peaks are less pronounced, and the valleys are shallower, indicating significantly better corrosion resistance. Even after 10 days, the surface remains relatively smooth with minimal roughness, suggesting that the 0.05 wt.% Sr modification offers the best protection against sulphuric acid corrosion, maintaining a smoother surface over an extended period.

Overall, the 3D profiles clearly demonstrate that the addition of Sr, particularly at 0.05 wt.%, significantly enhances the corrosion resistance of the LM6 alloy when immersed in sulphuric acid, as evidenced by the smoother surface topographies and reduced roughness in the modified samples.

**Figure 5:**
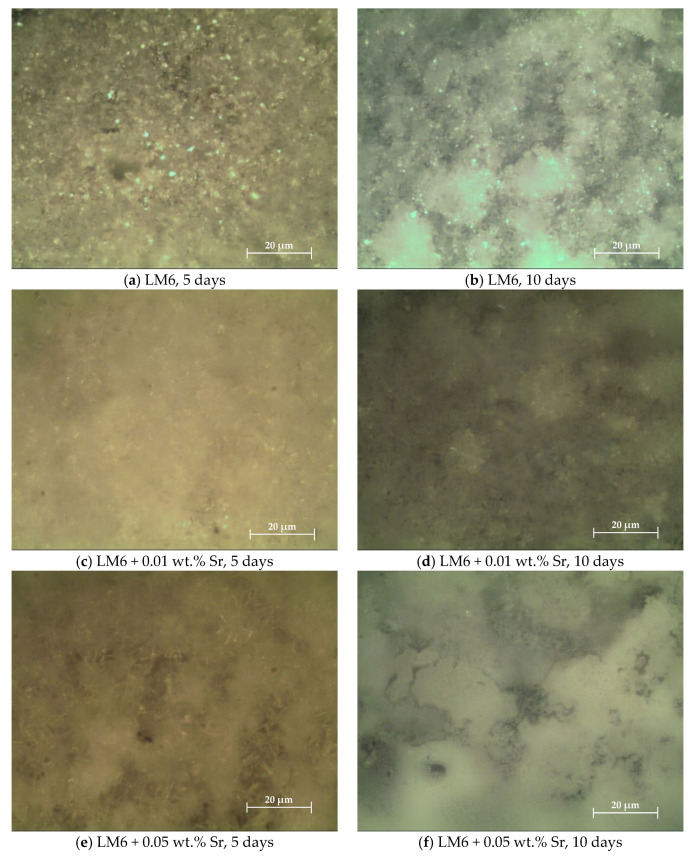
Morphology in 20× magnification immersed in sodium hydroxide. (**a**) LM6, 5 days; (**b**) LM6, 10 days; (**c**) LM6 + 0.01 wt.% Sr, 5 days; (**d**) LM6 + 0.01 wt.% Sr, 10 days; (**e**) LM6 + 0.05 wt.% Sr, 5 days; and (**f**) LM6 + 0.05 wt.% Sr, 10 days.

Figure 5a,b show significant surface degradation and roughness in the unmodified LM6 alloy after immersion in sodium hydroxide. The surface exhibits pronounced pits, indicating severe corrosion, with visible signs of degradation. Compared to the 5-day immersion, surface roughness increases further after 10 days, with larger pits and more pronounced surface degradation, highlighting the extensive corrosive effect of sodium hydroxide on the alloy.

Figure 5c,d demonstrate improved surface morphology for the alloy modified with 0.01 wt.% Sr, showing reduced roughness and fewer pits. This indicates enhanced corrosion resistance compared to the unmodified LM6. While there is still some increase in surface roughness after 10 days, the level of degradation remains less severe, illustrating the beneficial impact of 0.01 wt.% Sr in improving the alloy’s resistance to sodium hydroxide corrosion.

Figure 5e,f exhibit the least surface roughness and minimal pitting for the alloy modified with 0.05 wt.% Sr, indicating the highest level of corrosion resistance among all samples. The surface is significantly smoother, with minimal pits and considerably reduced roughness, showcasing excellent corrosion resistance. Even after extended exposure to sodium hydroxide, the 0.05 wt.% Sr modification maintains a relatively smooth surface with only minor roughness and fewer, less severe pits. This demonstrates the superior performance of the 0.05 wt.% Sr addition in resisting corrosion.

**Figure 6:**
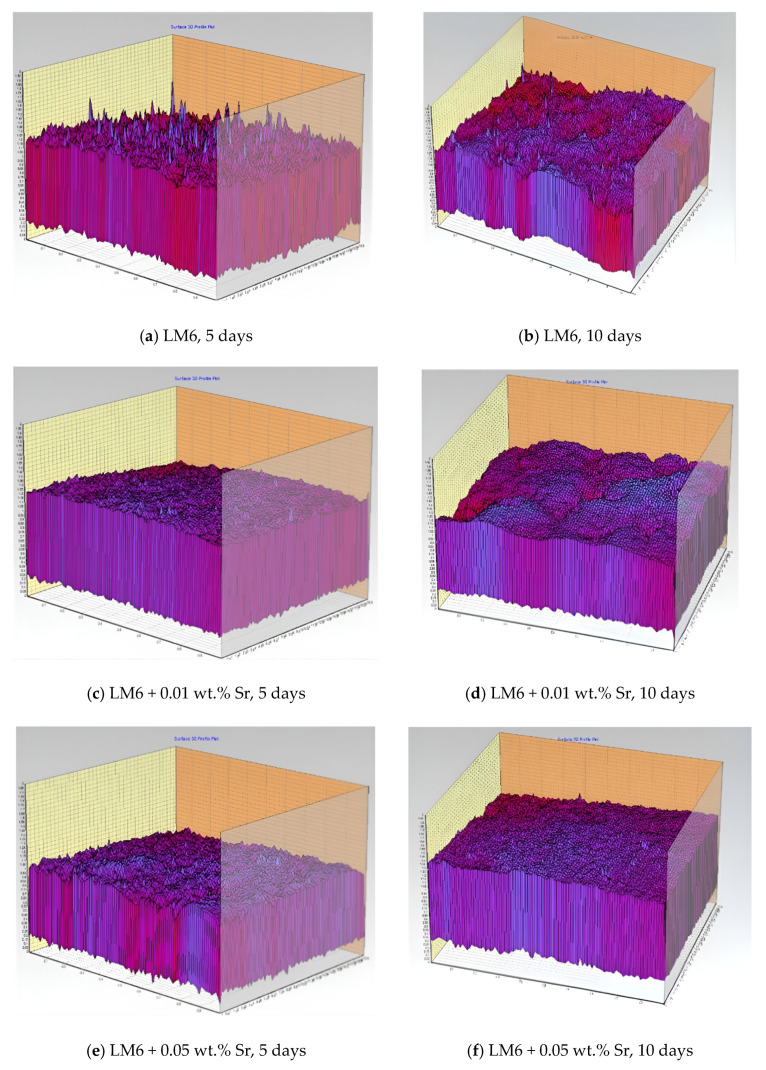
Three-dimensional profiles in 20× magnification immersed in sodium hydroxide. (**a**) LM6, 5 days; (**b**) LM6, 10 days; (**c**) LM6 + 0.01 wt.% Sr, 5 days; (**d**) LM6 + 0.01 wt.% Sr, 10 days; (**e**) LM6 + 0.05 wt.% Sr, 5 days; and (**f**) LM6 + 0.05 wt.% Sr, 10 days.

The unmodified LM6 alloy exhibits significant surface roughness and irregularity after immersion in sodium hydroxide, as depicted in Figure 6a,b. After 5 days, numerous peaks and valleys indicate considerable surface degradation. After 10 days, the surface roughness increases further, with more pronounced peaks and valleys, suggesting severe corrosion over the extended period. The surface shows significant roughness and irregularity, with numerous peaks and valleys. This indicates a considerable degree of surface degradation due to the corrosive environment of the sodium hydroxide. The surface roughness and irregularity have increased compared to the 5-day immersion. The peaks and valleys are more pronounced, suggesting continued and severe corrosion over the extended period.

The LM6 alloy with 0.01 wt.% Sr addition in Figure 6c,d shows reduced surface roughness compared to the unmodified LM6. After 5 days, the surface is smoother with less pronounced peaks and valleys, indicating mitigated corrosion effects. After 10 days, while there is an increase in surface roughness compared to the 5-day immersion, the corrosion effects are still less severe than those seen in the unmodified LM6, demonstrating improved corrosion resistance due to the addition of strontium. The surface appears smoother than the unmodified LM6 after the same immersion period. The peaks and valleys are less pronounced, indicating that the addition of strontium has somewhat mitigated the corrosion effects. The surface remains relatively smoother compared to the unmodified LM6 after 10 days, though there is an increase in roughness compared to the 5-day immersion. This suggests that while Sr addition reduces corrosion, prolonged exposure still leads to degradation.

The LM6 alloy with 0.05 wt.% Sr addition in Figure 6e,f exhibits the smoothest surface among the samples. After 5 days, the surface shows significantly fewer and less pronounced peaks and valleys, indicating high effectiveness in reducing corrosion effects. After 10 days, although there is some increase in surface roughness, it remains less severe compared to both the unmodified LM6 and the alloy with 0.01 wt.% Sr, demonstrating that 0.05 wt.% Sr provides the best corrosion resistance over prolonged exposure. The surface is notably smoother than both the unmodified LM6 and the alloy with 0.01 wt.% Sr after 5 days. This indicates a higher effectiveness of 0.05 wt.% Sr in reducing corrosion effects, with fewer and less pronounced peaks and valleys. The surface remains smoother compared to the unmodified LM6 and the alloy with 0.01 wt.% Sr after 10 days. The corrosion effects are less severe, with fewer significant peaks and valleys, demonstrating that 0.05 wt.% Sr provides better corrosion resistance over prolonged exposure.

The 3D profiles in Figure 6a–f indicate that the addition of Sr to the LM6 alloy reduces surface roughness and corrosion effects when immersed in sodium hydroxide. The higher concentration of strontium (0.05 wt.%) shows a more significant reduction in surface degradation compared to the lower concentration (0.01 wt.%). Prolonged immersion periods increase surface roughness and corrosion in all samples, but the strontium-modified alloys exhibit better resistance than the unmodified LM6 alloy.

**Figure 7:**
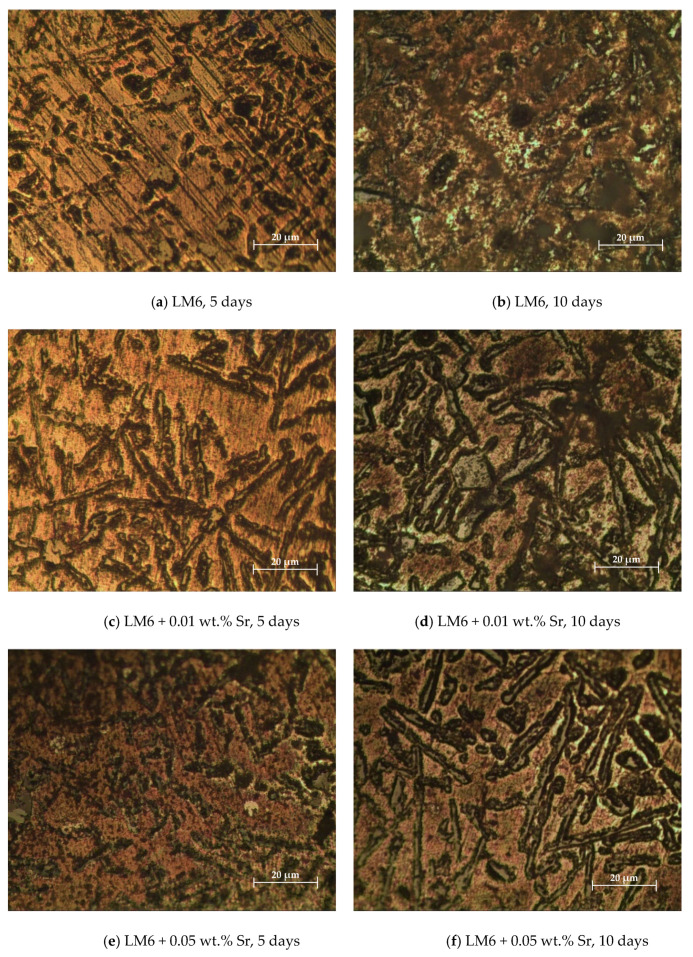
Morphology in 20× magnification immersed in 3.5% sodium chloride solution. (**a**) LM6, 5 days; (**b**) LM6, 10 days; (**c**) LM6 + 0.01 wt.% Sr, 5 days; (**d**) LM6 + 0.01 wt.% Sr, 10 days; (**e**) LM6 + 0.05 wt.% Sr, 5 days; and (**f**) LM6 + 0.05 wt.% Sr, 10 days.

The unmodified LM6 shows severe corrosion with significant surface degradation and pitting after 5 and 10 days, in Figure 7a,b. The unmodified LM6 surface after 5 days of immersion in 3.5% sodium chloride solution exhibits signs of corrosion. The morphology shows the development of corrosion products and pitting. The surface appears rough with clear indications of material degradation. After 10 days, the extent of corrosion is significantly higher. The surface morphology displays more extensive corrosion products and deeper pits. The surface roughness has increased, indicating ongoing and severe corrosion processes.

LM6 + 0.01 wt.% Sr demonstrates improved corrosion resistance with less surface roughness and fewer corrosion products in Figure 7c,d compared to the unmodified LM6, though degradation increases over time. The addition of 0.01 wt.% Sr to LM6 results in noticeable improvements in corrosion resistance. After 5 days, the surface shows fewer corrosion products and less pitting compared to the unmodified LM6. The overall surface roughness is reduced, indicating better resistance to the corrosive environment. After 10 days, the corrosion is more evident, but the severity is still less than that observed in the unmodified LM6. The surface displays more corrosion products and pitting than after 5 days, but the overall degradation is controlled, suggesting that 0.01 wt.% Sr provides moderate protection against sodium chloride-induced corrosion.

LM6 + 0.05 wt.% Sr exhibits the best corrosion resistance, with the smoothest surface and minimal corrosion products after both 5 and 10 days, indicating effective protection against the corrosive environment as shown in Figure 7e,f. The LM6 alloy with 0.05 wt.% Sr addition shows the least amount of corrosion after 5 days. The surface morphology is significantly smoother with minimal corrosion products and pitting. This indicates a high level of corrosion resistance, with the strontium effectively mitigating the corrosive effects of the sodium chloride solution. Even after 10 days, the LM6 alloy with 0.05 wt.% Sr maintains its integrity better than the other compositions. While there is some increase in surface roughness and corrosion products, the extent of corrosion is minimal compared to both the unmodified LM6 and the alloy with 0.01 wt.% Sr. This demonstrates that 0.05 wt.% Sr offers the best protection against prolonged exposure to the sodium chloride solution.

**Figure 8:**
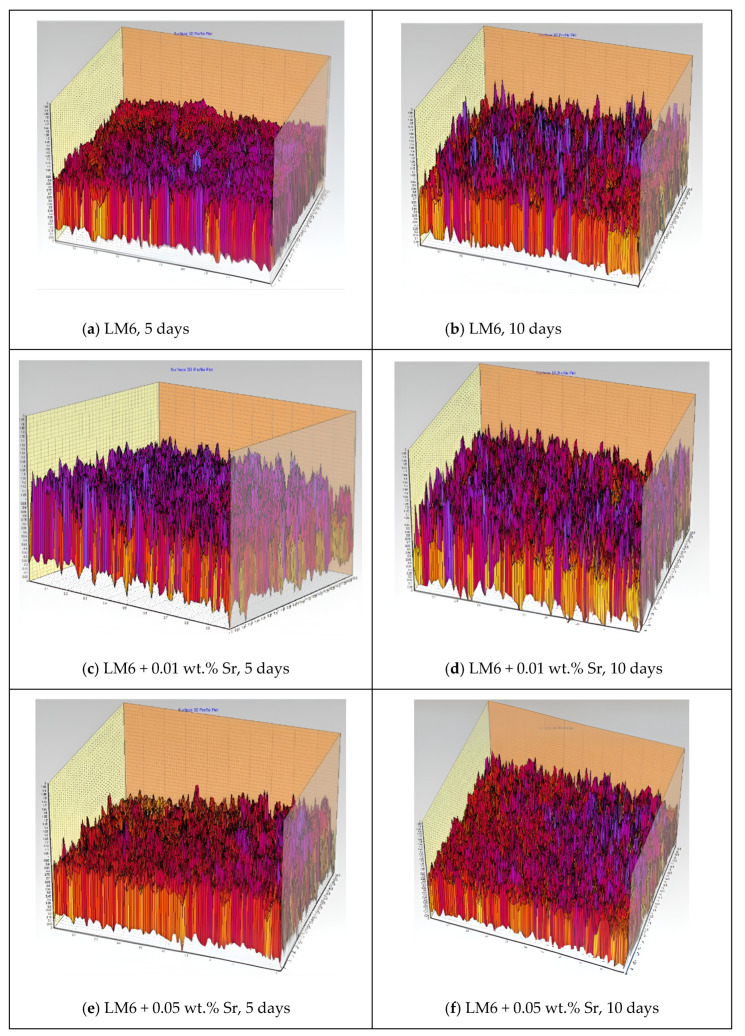
Three-dimensional profiles in 20× magnification immersed in 3.5% sodium chloride solution. (**a**) LM6, 5 days; (**b**) LM6, 10 days; (**c**) LM6 + 0.01 wt.% Sr, 5 days; (**d**) LM6 + 0.01 wt.% Sr, 10 days; (**e**) LM6 + 0.05 wt.% Sr, 5 days; and (**f**) LM6 + 0.05 wt.% Sr, 10 days.

Figure 8a,b show significant surface roughness and pronounced corrosion after 5 and 10 days, with deep pits and extensive corrosion products. The 3D profile of the unmodified LM6 surface after 5 days of immersion in 3.5% sodium chloride solution shows significant surface roughness and irregularities. The peaks and valleys indicate the presence of corrosion products and pitting, suggesting that the alloy is undergoing a substantial corrosive attack. After 10 days, the unmodified LM6 surface exhibits even more pronounced surface roughness. The 3D profile shows deeper pits and more extensive corrosion products compared to the 5-day immersion. This indicates an ongoing and severe corrosion process.

Figure 8c,d demonstrate improved corrosion resistance with a smoother surface and fewer pits. The surface roughness increases over time, but the extent of corrosion is still controlled compared to the unmodified LM6. The addition of 0.01 wt.% Sr to LM6 shows a notable improvement in the surface profile after 5 days. The 3D profile reveals a smoother surface with fewer and shallower pits. The presence of Sr appears to have mitigated the corrosion process to some extent. After 10 days, the surface of LM6 with 0.01 wt.% Sr still exhibits less corrosion than the unmodified LM6, but the roughness has increased compared to the 5-day immersion. The 3D profile shows more pronounced peaks and valleys, indicating the continued but controlled progression of corrosion.

In Figure 8e,f, LM6 + 0.05 wt.% Sr exhibits the best corrosion resistance, with the smoothest surface profile and minimal corrosion even after 10 days. This Sr concentration effectively mitigates the corrosive effects of the sodium chloride solution, maintaining surface integrity over time. The LM6 alloy with 0.05 wt.% Sr addition shows the least amount of surface roughness after 5 days. The 3D profile is significantly smoother with minimal peaks and valleys, suggesting that this Sr concentration offers superior protection against the corrosive environment. Even after 10 days, the LM6 alloy with 0.05 wt.% Sr maintains a relatively smooth surface profile. The 3D profile shows only slight increases in roughness compared to the 5-day immersion. This indicates that 0.05 wt.% Sr provides effective and sustained protection against sodium chloride-induced corrosion over time.

Table 2, Table 3 and Table 4 present the corrosion testing results of LM6 and its modified variants (with 0.01 wt.% Sr and 0.05 wt.% Sr) when immersed in sulphuric acid, sodium hydroxide, and 3.5% sodium chloride solution over 5 and 10 days. Unmodified LM6 exhibits significant weight loss in both sulphuric acid and sodium hydroxide, indicating high corrosion susceptibility, but shows minimal weight change in 3.5% saltwater, suggesting good resistance. LM6 alloyed with 0.01 wt.% Sr shows a slight reduction in weight loss in sulphuric acid but an increased weight loss in sodium hydroxide, indicating higher susceptibility to this agent, while maintaining resistance to 3.5% sodium chloride solution similar to unmodified LM6. In contrast, LM6 with 0.05 wt.% Sr displays reduced weight loss in sulphuric acid but demonstrates the highest susceptibility to sodium hydroxide, evidenced by the significant weight loss. This variant also maintains good resistance to 3.5% sodium chloride solution, with minimal weight changes observed. Overall, the addition of Sr modifies the corrosion behaviour, with notable variations depending on the corrosive environment and Sr content.

**Figure 9:**
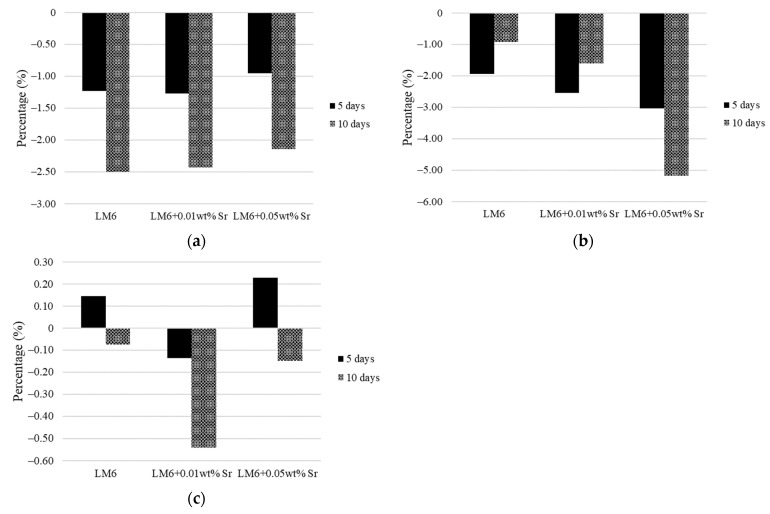
Weight-reduced percentage of LM6 and modified LM6 immersed in (**a**) sulphuric acid, (**b**) sodium hydroxide, and (**c**) 3.5% of sodium chloride solution.

Figure 9a–c illustrate that the corrosion resistance of the LM6 alloys is dependent on the corrosive environment, with LM6 + 0.05 wt.% Sr generally showing better performance in sulphuric acid and sodium chloride solution, but not in sodium hydroxide. The results in Figure 9c show some increases in the weight of LM6 and its modified variants immersed in a 3.5% sodium chloride solution. This increment can be attributed to the formation of corrosion products on the surface of the alloys. When aluminium alloys are exposed to chloride solutions, corrosion can occur, leading to the formation of aluminium chloride and other corrosion by-products. These by-products can adhere to the surface of the alloy, effectively increasing its weight. Additionally, the presence of Sr in the alloy can influence the nature and amount of these corrosion products, resulting in variations in the observed weight changes.

### 3.3. Corrosion Rate

**Figure 10:**
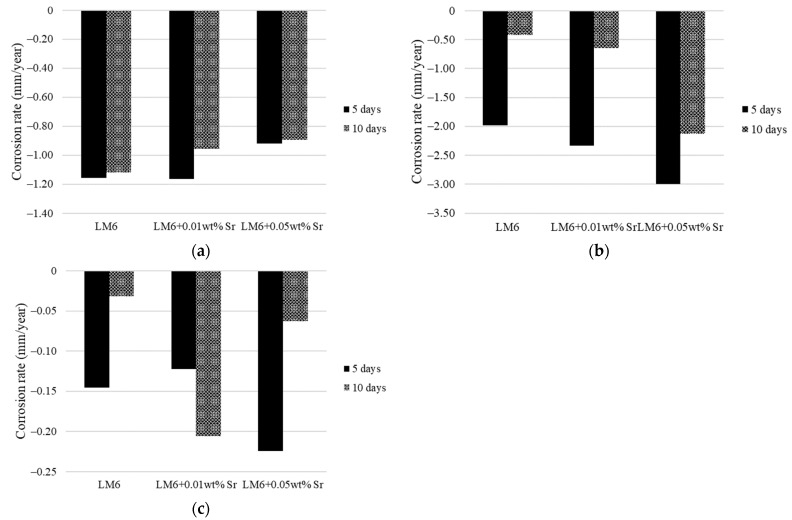
Corrosion rates of LM6 and modified LM6 in (**a**) sulphuric acid, (**b**) sodium hydroxide, and (**c**) 3.5% of sodium chloride solution.

Table 5 and Figure 10a–c present the corrosion rates of unmodified LM6 and LM6 modified with 0.01 wt.% Sr and 0.05 wt.% Sr in various corrosive environments over 5 and 10 days. In sulphuric acid, LM6 with 0.05 wt.% Sr shows the lowest corrosion rates, indicating enhanced resistance compared to the unmodified alloy and the 0.01 wt.% Sr variant. However, in sodium hydroxide, both Sr-modified alloys exhibit higher corrosion rates than the unmodified LM6, with the 0.05 wt.% Sr modification showing the highest rates, suggesting reduced resistance in this environment. In the 3.5% sodium chloride solution, the corrosion rates for all specimens are relatively low, with the 0.05 wt.% Sr alloy initially showing a higher rate but reducing over time, indicating good overall resistance. These observations highlight that while Sr additions can improve corrosion resistance in certain environments like sulphuric acid and 3.5% sodium chloride solution, they may significantly increase susceptibility to corrosion in sodium hydroxide.

The corrosion mechanisms observed in this study reveal that in sulphuric acid, aluminium initially forms a passive oxide layer (Al_2_O_3_) that acts as a protective barrier. The addition of strontium (Sr) enhances this passive layer, significantly reducing corrosion rates, as confirmed by weight loss measurements. In sodium hydroxide, the passive oxide layer is aggressively attacked and dissolved, causing uniform corrosion. While the Sr addition mitigates some dissolution, uniform corrosion remains predominant, as evidenced by the even distribution of corrosion products. In the sodium chloride solution, pitting corrosion occurs as chloride ions penetrate the passive oxide layer. Sr improves the integrity of the passive layer, reducing pit density and depth, as shown by surface analysis and weight loss data. The enhanced corrosion resistance of the LM6 (A413) aluminium alloy due to Sr is attributed to two key mechanisms: microstructural refinement and the formation of a stable passive oxide layer. Sr refines the coarse eutectic silicon phase into a finer, fibrous, or lamellar structure, which reduces localised corrosion initiation sites and stress concentrations. This refinement also minimizes galvanic coupling between the silicon and aluminium matrix, lowering the electrochemical potential difference that drives corrosion. Moreover, Sr promotes the formation of a more stable passive oxide layer, providing better protection against corrosive ion penetration, especially in aggressive environments like sulphuric acid and sodium chloride. In sodium hydroxide, Sr reduces uniform corrosion by slowing the dissolution of the aluminium matrix. As a result, the refined microstructure and enhanced passive layer significantly improve the overall corrosion resistance of Sr-modified LM6 alloys.

Here are the key reactions that describe the mechanism affecting corrosion for the LM6 (A413) aluminium alloy in various corrosive environments:

When aluminium reacts with sulphuric acid, it undergoes dissolution and forms aluminium sulphate, while releasing hydrogen gas. The reaction can be represented as follows:

2Al + 3H_2_SO_4_ → Al_2_(SO_4_)_3_ + 3H_2_↑

At the same time, a protective aluminium oxide layer (Al_2_O_3_) may form as follows:

4Al + 3O_2_ → 2Al_2_O_3_

The formation of this oxide layer slows down further corrosion, but if the acid is aggressive, the oxide layer can be dissolved, leading to more corrosion.

In the presence of sodium hydroxide, aluminium reacts to form sodium aluminate, and hydrogen gas is released as follows

2Al + 6NaOH → 2Na_3_AlO_3_ + 3H_2_↑

Here, sodium hydroxide dissolves the protective oxide layer, leading to uniform corrosion of the aluminium surface.

Sodium chloride leads to pitting corrosion, where chloride ions (Cl^−^) penetrate the passive aluminium oxide layer, initiating localised corrosion. The reaction for pitting initiation is as follows:

Al + 3Cl^−^ → AlCl_3_ + 3e^−^

As the chloride ions break down the oxide layer, pitting occurs, which propagates rapidly, leading to localised damage.

These equations represent the electrochemical reactions that occur in sulphuric acid, sodium hydroxide, and sodium chloride solutions, affecting the corrosion behaviour of the LM6 alloy. The presence of strontium (Sr) helps in forming a more stable oxide layer, reducing the aggressiveness of these reactions.

## 4. Conclusions

The incorporation of strontium (Sr) into an LM6 (A413) aluminium alloy significantly enhances its corrosion resistance across diverse corrosive environments. This study demonstrates that the addition of Sr, particularly at concentrations of 0.01 wt.% and 0.05 wt.%, effectively refines the alloy’s microstructure, transforming the eutectic silicon phase into a more desirable fibrous or lamellar structure. This microstructural refinement not only improves surface morphology but also results in a substantial reduction in corrosion rates. The 0.05 wt.% Sr concentration shows the greatest overall enhancement, providing superior resistance to sulphuric acid, sodium hydroxide, and sodium chloride solutions, with up to a 5% reduction in weight loss observed in aggressive alkaline conditions. The formation of stable passive oxide layers further contributes to the enhanced corrosion resistance in acidic environments. These findings underscore the potential of Sr as a powerful microstructural modifier, offering significant improvements in both the performance and longevity of Al-Si alloys in industrial applications, where corrosion resistance is critical.

## Figures and Tables

**Table 1:** Chemical composition of LM6 (A413) alloy in wt.%.

Alloy	Al	Si	Fe	Cu	Mn	Mg	Zn	Ti
LM6 (A413)	Balance	12.0	0.6	0.1	0.5	0.1	0.1	0.2

**Table 2:** LM6 corrosion testing.

Corrosion Agent	Days of Immersion (Days)	Original Weight (g)	Final Weight (g)	Weight Change (g)	Change in Percentage (%)
Sulphuric acid	5	1.379	1.362	−0.017	−1.233
Sulphuric acid	10	1.399	1.364	−0.035	−2.502
Sodium hydroxide	5	1.399	1.372	−0.027	−1.930
Sodium hydroxide	10	1.413	1.400	−0.013	−0.920
3.5% Sodium chloride solution	5	1.372	1.374	0.002	0.146
3.5% Sodium chloride solution	10	1.364	1.363	−0.001	−0.073

**Table 3:** LM6 + 0.01 wt.% Sr corrosion testing.

Corrosion Agent	Days of Immersion (Days)	Original Weight (g)	Final Weight (g)	Weight Change (g)	Change in Percentage (%)
Sulphuric acid	5	1.421	1.403	−0.018	−1.267
Sulphuric acid	10	1.401	1.367	−0.034	−2.427
Sodium hydroxide	5	1.455	1.418	−0.037	−2.543
Sodium hydroxide	10	1.438	1.415	−0.023	−1.599
3.5% Sodium chloride solution	5	1.463	1.461	−0.002	−0.137
3.5% Sodium chloride solution	10	1.483	1.475	−0.008	−0.539

**Table 4:** LM6 + 0.05 wt.% Sr corrosion testing.

Corrosion Agent	Days of Immersion (Days)	Original Weight (g)	Final Weight (g)	Weight Change (g)	Change in Percentage (%)
Sulphuric acid	5	1.368	1.355	−0.013	−0.950
Sulphuric acid	10	1.355	1.326	−0.029	−2.140
Sodium hydroxide	5	1.388	1.346	−0.042	−3.026
Sodium hydroxide	10	1.237	1.173	−0.064	−5.174
3.5% Sodium chloride solution	5	1.315	1.318	0.003	0.228
3.5% Sodium chloride solution	10	1.344	1.342	−0.002	−0.149

**Table 5:** Corrosion rates of different specimens.

Specimen	Sulphuric Acid		Sodium Hydroxide		Saltwater	
	5 Days	10 Days	5 Days	10 Days	5 Days	10 Days
LM6	−1.1555	−1.1176	−1.9825	−0.4200	0.1455	−0.0316
LM6 + 0.01 wt% Sr	−1.1615	−0.9560	−2.3335	−0.6482	−0.1225	−0.2058
LM6 + 0.05 wt% Sr	−0.9171	−0.8945	−2.9916	−2.1257	0.2240	−0.0630

## Data Availability

The original contributions presented in the study are included in the article, further inquiries can be directed to the corresponding author.
